# Fusion expression of nanobodies specific for the insecticide fipronil on magnetosomes in *Magnetospirillum gryphiswaldense* MSR-1

**DOI:** 10.1186/s12951-021-00773-z

**Published:** 2021-01-19

**Authors:** Sha Wu, Fengfei Ma, Jinxin He, Qing X. Li, Bruce D. Hammock, Jiesheng Tian, Ting Xu

**Affiliations:** 1 Beijing Key Laboratory of Biodiversity and Organic Farming, College of Resources and Environmental Sciences, China Agricultural University, Beijing, 100193 China; 2 Suzhou Vicheck Biotechnology Co. Ltd, Suzhou, 215128 China; 3 Department of Molecular Biosciences and Bioengineering, University of Hawaii At Manoa, 1955 East-West Road, Honolulu, HI 96822 USA; 4 Department of Entomology and Nematology and UCD Comprehensive Cancer Center, University of California, Davis, CA 95616 USA; 5 Department of Microbiology, College of Biological Sciences, China Agricultural University, Beijing, 100193 China

**Keywords:** Magnetosome, *Magnetospirillum gryphiswaldense*, Nanobody, Fipronil, Immunoassay

## Abstract

**Background:**

Magnetic nanoparticles such as magnetosomes modified with antibodies allow a high probability of their interaction with targets of interest. Magnetosomes biomineralized by magnetotactic bacteria are in homogeneous nanoscale size and have crystallographic structure, and high thermal and colloidal stability. Camelidae derived nanobodies (Nbs) are small in size, thermal stable, highly water soluble, easy to produce, and fusible with magnetosomes. We aimed to functionalize Nb-magnetosomes for the analysis of the insecticide fipronil.

**Results:**

Three recombinant magnetotactic bacteria (CF, CF+ , and CFFF) biomineralizing magnetosomes with different abundance of Nbs displayed on the surface were constructed. Compared to magnetosomes from the wild type *Magnetospirillum gryphiswaldense* MSR-1, all of the Nb-magnetosomes biosynthesized by strains CF, CF+ , and CFFF showed a detectable level of binding capability to fipronil-horseradish peroxidase (H2-HRP), but none of them recognized free fipronil. The Nb-magnetosomes from CFFF were oxidized with H_2_O_2_ or a glutathione mixture consisting of reduced glutathione and oxidized glutathione in vitro and their binding affinity to H2-HRP was decreased, whereas that to free fipronil was enhanced. The magnetosomes treated with the glutathione mixture were employed to develop an enzyme-linked immunosorbent assay for the detection of fipronil in water samples, with average recoveries in a range of 78–101%.

**Conclusions:**

The economical and environmental-friendly Nb-magnetosomes biomineralized by the bacterial strain MSR-1 can be potentially applied to nanobody-based immunoassays for the detection of fipronil or nanobody-based assays in general.
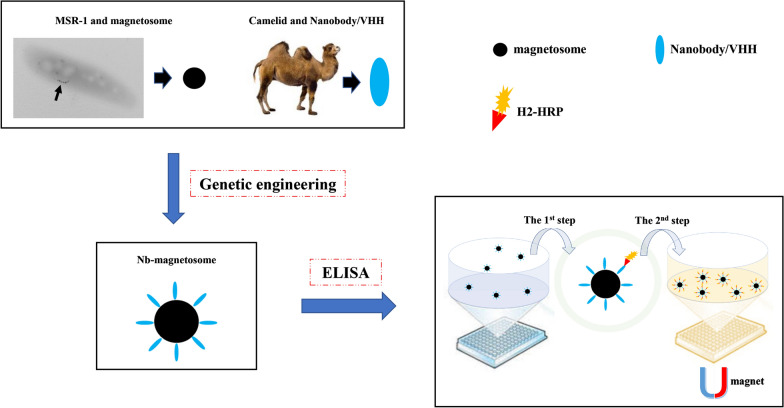

## Background

Magnetic nanoparticles (MNPs) have been extensively applied in the life sciences due to their multiple attributes such as large surface-area-to-volume ratio, biocompatibility and simple separation methodology [1, 2]. Magnetite (Fe_3_O_4_), one of the magnetic materials widely used in MNPs, are usually produced by co-precipitation as the simplest and most economical method [3]. A variety of coating materials (e.g., chitosan) of MNP surfaces have been used to reduce the aggregation of particles, preserve their stability and facilitate their further functionalization with biomolecules [4, 5].

In contrast to MNPs produced by a physical–chemical reaction, magnetosomes are biomineralized by magnetotactic bacteria (MTB), a phylogenetically and physiologically diverse group of prokaryotes [6]. Magnetosomes consisting of magnetite materials (Fe_3_O_4_) covered by a bilayer membrane with numerous specific proteins have advantages over other MNPs for their homogeneous sizes and crystallography as well as high thermal and colloidal stability [7, 8]. Magnetosomes functionalized with genetic or chemical methods have gained considerable interest in a broad range of applications, such as immunoassays, biosensor, drug delivery and biomedical imaging [8]. Because of their cost-effective and environmental-friendly nature, genetic modifications to functionalize magnetosomes have been an important research topic. Many of the proteins in magnetosome membranes have been studied to uncover their roles in magnetosome biomineralization [7, 9]. This fundamental knowledge has been used to modify magnetosomes [8, 10]. MamC and MamF are acknowledged as two of the most abundant proteins in magnetosome membrane. They are relatively small proteins (~ 12.5 kD) with two or three predicted transmembrane helices associated tightly with the magnetosome membrane [9]. *mamC* and *mamF* are both in the *mamGFDC* operon of *Magnetospirillum gryphiswaldense* MSR-1, and have a minor and nonessential function in magnetite biomineralization [11]. Thus, MamC and MamF have been frequently exploited for the magnetosomal display of functional proteins such as immunoglobulin G-binding domains [12], thyroid-stimulating hormone receptor (TSHR) [13] and Staphylococcal protein A (SPA) [14].

The variable domain of Camelidae heavy-chain-only antibodies (VHHs), also referred to as nanobodies (Nbs), have superior properties to conventional antibodies for their small size, non-immunogenicity, thermal stability, high solubility, ease of production in microorganisms and ease of genetic modification [15, 16]. The extensive availability of molecular biological techniques facilitates the genetic conjugation of Nbs to magnetosomes, but only one investigation on the fusion expression of Nbs binding red fluorescent protein (RFP) on magnetosomes has been reported so far [17]. Over the past few years, Nbs are attractive in the field of immunoassays for monitoring small molecules such as biomarkers and environmental pollutants [18]. Nbs chemically conjugated to magnetosomes proved to be an effective tool for the detection of environmental compounds [19, 20]. It is promising to display nanobodies on magnetosomes by a genetic method to monitor small-molecule contaminants.

In our previous study, Nbs selective to fipronil and its metabolites were generated and used for the detection of fipronil in rodent sera [21]. Fipronil is the first phenylpyrazole insecticide that acts at the γ-aminobutyric acid (GABA) receptor of insects, blocking the passage of chloride ions [22]. It is widely used for the control of field insects in agriculture as well as fleas and ticks on pets [23]. Nevertheless, the widespread use of fipronil has induced an increasing concern about the possible off-target harm to ecosystems and human health particularly when employed in non approved application [24].

Here, we displayed a Nb (F1) on magnetosomes by gene fusion using MamC and MamF as anchoring proteins in MSR-1. MSR-1 gives the highest magnetosome yields among the MTB [25], to construct functional MNPs specific for fipronil. The modified magnetosomes could be easily produced with low cost, having a potential application in the field of immunoassays.

## Methods and materials

### Bacteria strains and culture conditions

The bacteria strains and plasmids used in this study are listed in Additional file 1: Table S1. *Escherichia coli* strains were cultured in Luria broth (LB) at 37 °C. MSR-1 was cultured in sodium lactate medium (SLM) or sodium glutamate medium (SGM) (substitute 4 g sodium glutamate for NH_4_Cl and yeast extract in SLM) as described previously [26]. Large-scale MSR-1 cells were fed-batch cultured in a 7.5 L-fermenter according to the method reported by Zhang et al. [27]. Antibiotics prepared in culture media were as follows: for *E. coli*, ampicillin at 100 μg/mL and kanamycin at 50 μg/mL; for MSR-1, kanamycin at 5 μg/mL and nalidixic acid at 5 μg/mL. The growth of MSR-1 (OD_565_) and the response to a magnet field (Cmag) were detected according to the methods described previously [28].

### Construction of recombinant plasmids and strains

All primers used in this study are listed in Additional file 1: Table S2. The restriction enzyme digestion (TaKaRa, Japan), DNA ligation (TaKaRa, Japan), and polymerase chain reaction (PCR) (Vazyme, China) were conducted according to manufacturers’ instructions. *mamC* and *mamF*, along with their upstream and downstream flanking sequences, were amplified from MSR-1 genomic DNA. The anti-fipronil Nb gene was amplified from Nb F1 encoded in the pComb 3X vector [21]. uCFd, a cassette consisting of *mamC* upstream region, *mamC*, Nb gene, and *mamC* downstream region, and uFFd, a cassette consisting of *mamF* upstream region, *mamF*, Nb gene, and *mamF* downstream region, were assembled by fusing PCR amplification. uCFd and uFFd were then subcloned into pK18mobSacB digested with *EcoR* I and *Xba* I to construct the suicide recombinant plasmids pKCF and pKFF, respectively. CF, a fusion gene consisting of *mamC* and Nb gene, was amplified from uCFd with primers MamC-F (*EcoR* I) and Fip-R (*Xba* I), and subcloned into pBBR1MCS-2 to create pBBRCF.

pKCF was transferred to wild type (WT) MSR-1 to generate a double crossover strain CF, which was successively cultured in medium containing kanamycin and 10% sucrose. pKFF and pBBRCF were then transferred to the strain CF to generate CFFF and CF + , respectively. Strains CF + and CFFF were cultured in medium containing kanamycin.

### Extraction of Nb-magnetosomes

Cells were harvested by centrifugation (8000×*g*). Approximately 1 g cell pellets were suspended in 40 mL of PBS (0.01 M phosphate, 0.137 M NaCl, and 0.003 M KCl, pH 7.4), which was prepared with ultrapure water and then autoclaved to denature protease and reduce the concentration of dissolved oxygen (dO_2_). The cells were ultrasonically disrupted under a power of 200 W for 30 min (work 3 s, stop 5 s every cycle), next to 100 W and 60 W in turn. The suspension of broken cells was placed in a magnetic field for 3.5 h to separate Nb-magnetosomes from solution. Precipitates were resuspended in PBS and washed ultrasonically (40 W) for about 30 min, followed by separation with magnetism. The suspend-wash-separate step was repeated until OD_260_/OD_280_ of supernatant proteins was unchanged. The extraction workflow is shown in Additional file 1: Fig. S1.

### Treatment of Nb-magnetosomes with oxidants

Nb-magnetosomes were treated with H_2_O_2_ solution (0.1–10%) or a mixture of reduced glutathione (GSH) and oxidized glutathione (GSSG) with different ratios (20:1, 10:1, 5:1, and 2:1). The treating time was 0.5, 1, 3, and 5 h.

### Couple of fipronil derivative to enzyme

The hapten (H2) (Additional file 1: Fig. S2) of fipronil was available from our previous study [21] and coupled to horseradish peroxidase (HRP) according to the method reported by Schneider and Hammock [29]. The concentrations of H2-HRP were determined with BCA protein assay.

### Nb-Magnetosome-based immunoassays for fipronil

Magnetosome-based enzyme-linked immunosorbent assays (ELISAs) for the analysis of small molecules were available from previous studies [19, 20]. Briefly, a 96-well microtiter plate was blocked with 1% gelatin dissolved in carbonate-bicarbonate buffer (pH 9.6) (300 μL per well) at 4 °C overnight, followed by washing with PBST (PBS containing 0.05% Tween-20) three times. Nb-magnetosomes were blocked with 2% bovine serum albumin (BSA) in PBS at room temperature for 3 h and washed with PBST. Afterwards, an aliquot volume of Nb-magnetosomes were transferred to the blocked microtiter plate. The solutions of fipronil and H2-HRP, each at 50 μL, were successively added into wells harboring Nb-magnetosomes, and the plate was incubated on an oscillator (150 rpm/min) at room temperature for 1 h. The plate was then fastened on a magnetic frame and washed with PBST. A 100-μL aliquot of 3,3′,5,5′-tetramethylbenzidine (TMB) solution (400 μL of 0.6% TMB and 100 μL of 1% H_2_O_2_ in 25 mL of citrate buffer, pH 5.5) was added into the wells and the reaction was stopped 10 min later by adding 50 μL of H_2_SO_4_ (2 M). Finally, the mixture was read at 450 nm on a microtiter plate reader (ELx800, Bio Tek, USA).

The resulting ELISA was applied to the analysis of fipronil in water samples which were collected from Lake Kunming in Beijing, China. Water samples were fortified with fipronil to reach the final concentrations of 100, 150, and 200 ng/mL. These samples were passed through a 0.22-μm filter (Waters, MA, USA) and then diluted with PBS (pH 7.4) prior to magnetosome-based ELISAs.

## Results

### Construction of recombinant strains to biomineralize Nb-magnetosomes

**Fig. 1:**
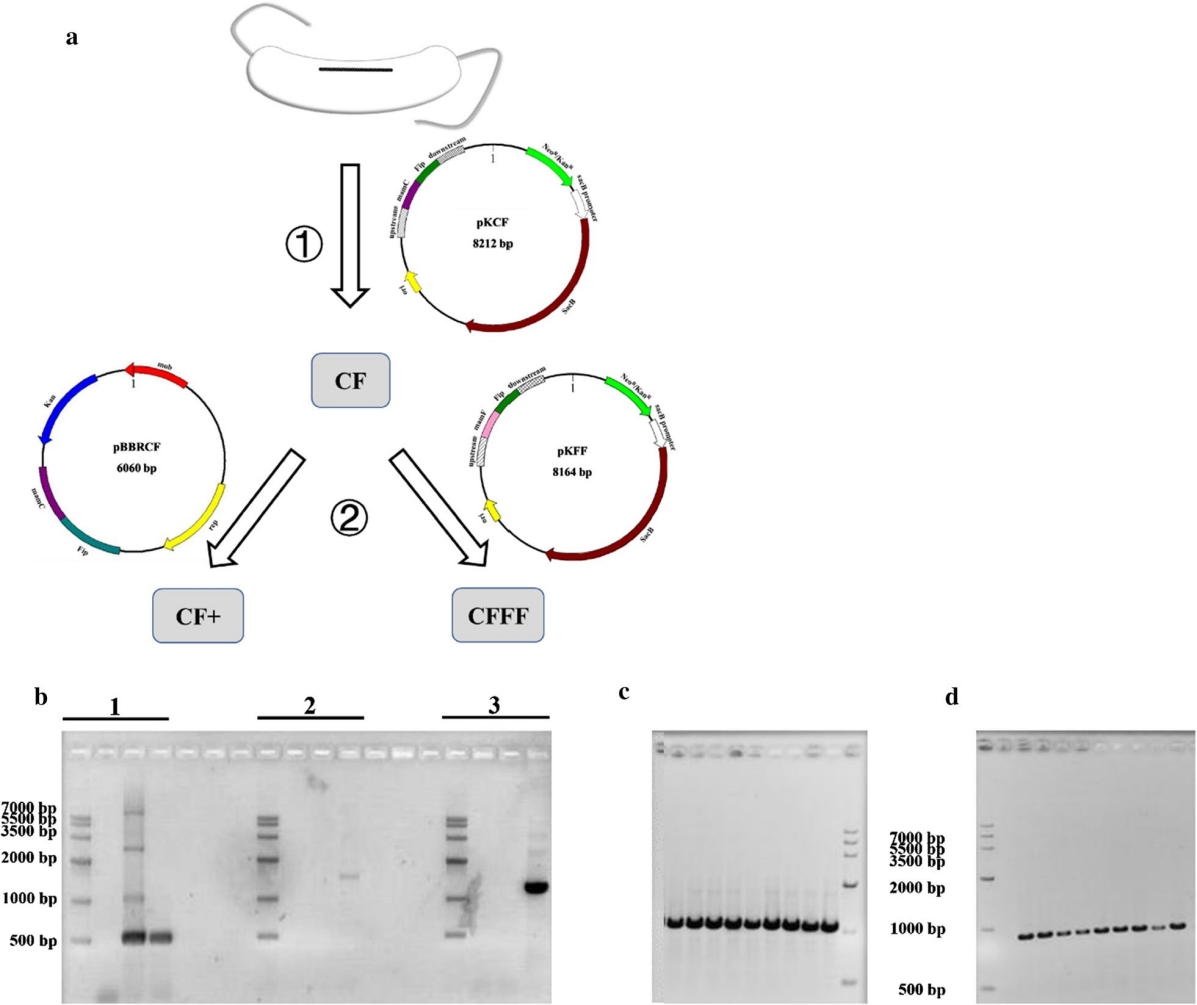
Construction of recombinant strains biomineralizing Nb-magnetosomes. **a** The schematic diagram of recombinant strain construction. **b** Colony PCR of CF. genes in group 1 were amplified by using Fip-F and Fip-R (left to right: marker, negative control, Nb F1, and CF); genes in group 2 were amplified by P1 and P3 (left to right: marker, negative control, WT MSR-1, and CF); genes in group 3 were amplified by P2 and P4 (left to right: marker, negative control, WT MSR-1, and CF). **c** Colony PCR of CF+ . The first lane and the last lane were negative control and marker, respectively; the rest of lanes were sampling. Primers used were pBBR-F and pBBR-R. **d** Colony PCR of CFFF. The first and the second lane were marker and negative control, respectively; the rest of lanes were sampling. Primers used were mamF and Fip-R. All primers were listed in Additional file 1: Table S2

MamC and MamF were selected as anchor proteins to engineer with anti-fipronil Nbs because they have high abundance in the membrane of the magnetosome from MSR-1 and negligible influence on the biomineralization of magnetosomes [9, 11]. In order to obtain Nb-magnetosomes possessing desirable affinity to fipronil, three recombinant strains that could biosynthesize magnetosomes with varying abundance of Nbs were constructed. The procedure of strain construction is shown in Fig. 1a.

Because *sacB*, a gene from *Bacillus subtilis,* encodes levansucrase coverting sucrose to levans, lethal to many Gram-negative bacteria (e.g., *Desulfovibrio magneticus* RS-1) [30], it is commonly used as a counterselection marker. A suicide vector, carrying a selectable marker flanked by upstream and downstream regions of homology to a host target gene, could be integrated into the chromosomes of hosts. The suicide vector pK18mobSacB has two features: (i) *neo* conferring resistances to neomycin and kanamycin can be used to confirm integration events at the designated site of genome; (ii) *sacB* assists in obtaining the desired mutant strains by preserving the target gene left in genome but excising its vector backbone from genome. In this way, the mutant strains can be further modified by inserting another pK18mobSacB or other plasmids. In the present study, pKCF carrying the gene cassette uCFd expressing the anti-fipronil Nb at the C-terminal of MamC was transferred into WT MSR-1 (Additional file 1: Tables S1 and S2) to generate a double crossover mutant strain, named as CF, which was successively identified by colony PCR (Fig. 1b) and gene sequencing. However, CF has only one copy of Nb F1 gene in its genome so that the expression of Nbs may be limited [31]. To increase the abundance of Nbs displaying on magnetosomes, a multi-copy broad host range vector pBBR1MCS-2 carrying a fusion gene CF expressing Nbs at the C-terminal of MamC, named as pBBRCF, was transferred into the mutant strain CF, generating CF+ with multi-copy Nb F1 gene, i.e., CF strain harbored plasmid pBBRCF in cytoplasm (Additional file 1: Table S1). In addition, a suicide vector pK18mobSacB carrying a gene cassette uFFd expressing Nbs at the C-terminal of MamF, named as pKFF, was conjugated to the mutant strain CF, generating CFFF with two copies of Nb F1 genes in MSR-1 genome, i.e., pKFF was integrated into CF strains’ chromosomes (Additional file 1: Table S2). Both CF+ and CFFF were identified via gene sequencing and colony PCR (Fig. 1c, d).

### Magnetic response of recombinant strains in fed-batch culture fermentation

**Fig. 2:**
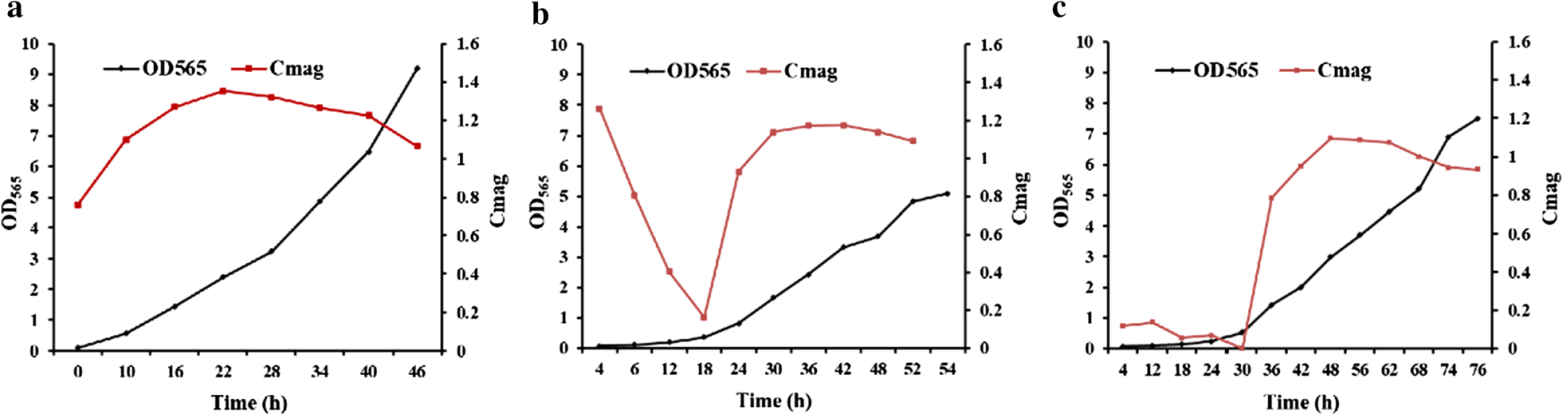
The growth (OD_565_) and magnetic response (Cmag values) curves of CF (**a**), CF + (**b**) and CFFF (**c**) fed-batch cultured in a fermenter

Each of the recombinant strains (CF, CF+ , and CFFF) was propagated three times (10% inoculation) in SLM supplemented with 20 μM ferric citrate, and then submerged in a 7.5-L fermenter for fed-batch culture. The lag phase of growth curves (OD_565_) from CF+ and CFFF was longer than that from CF (Fig. 2), suggesting that the growth and magnetosome biomineralization of strains would be restrained by transferring pBBRCF and pKFF into CF.

Cmag values were calculated by measuring the maximum and minimum scattering intensities [32]. A typical parabolic curve of Cmag values was observed from the strain CF (Fig. 2a), but the curves of Cmag values from CF+ and CFFF could be divided into two parts: a decreasing curve and a parabolic curve (Fig. 2b, c). It is well known that MSR-1 biosynthesizes magnetosomes under low oxygen conditions (dO_2_ < 1%). When cells were transferred into the fermenter, dO_2_ of the culture medium was high and the biomineralization of magnetosomes in cells was temporarily inhibited, leading to the initial decrease of Cmag from CF+ and CFFF. Nonetheless, CF grew faster than CF+ and CFFF, and dO_2_ could be quickly driven down to a concentration suitable for the biomineralization of magnetosomes in CF after the transfer. Therefore, the initial decrease curve of Cmag was not observed from CF. After the culture of CF, CF+ and CFFF for 22, 36, and 48 h, respectively, Cmag values declined steadily, illustrating that dO_2_ was enhanced with the increase of stirring rate. In general, the cells should be harvested before Cmag values dropped dramatically to ensure a sufficient yield of Nb-magnetosomes. Hence, when the Cmag values of CF, CF+ , and CFFF moved down to approximately 1.0 from the peak, cells were harvested even though they were still in the exponential phase of growth.

### Characteristics of Nb-magnetosomes

Nb-magnetosomes were extracted and purified ultrasonically under different power (Additional file 1: Fig. S1). As the concentration of proteins in the supernatant (OD_260_/OD_280_) was constant, the purification procedure was stopped to avoid the damage of magnetosome membranes.

**Fig. 3:**
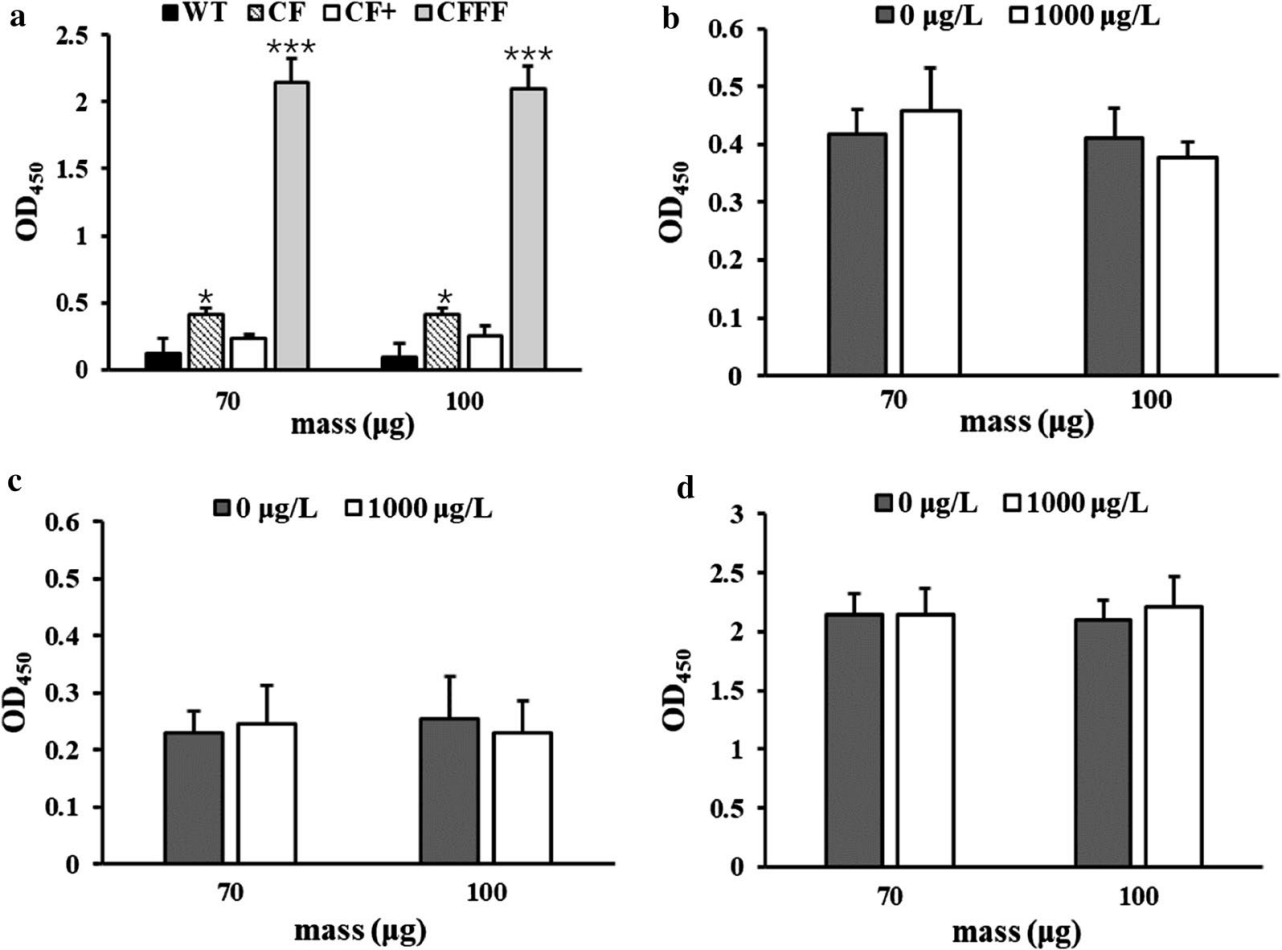
Non-competitive ELISAs based on three Nb-magnetosomes (**a)** and competitive ELISAs for fipronil based on Nb-magnetosomes from CF (**b**); CF+ (**c**) and CFFF (**d**). “*”: significant difference between WT and CF (*p* < 0.05); “***”: large significant difference between WT and CFFF (*p* < 0.01)

Nb-magnetosomes synthesized by CF, CF+ , and CFFF exhibited different binding capability to H2-HRP in a non-competitive ELISA, with the highest OD_450_ from CFFF (Fig. 3a). Compared to CF, CFFF with a suicide vector pKFF integrated into chromosomes probably expressed more Nbs on the surface of magnetosomes, but CF+ with a plasmid pBBRCF in cytoplasm did not show more Nb expression as expected. It was reported that in the construction of a complementary strain, gene copies, promoters, and regulatory processes were distinctive in plasmid vs genome structure, causing the difference of transcription and expression levels [33]. Unfortunately, none of the Nb-magnetosomes exhibited binding ability to fipronil at 1000 ng/mL in a competitive ELISA (Fig. 3b-d), suggesting that the binding affinity of Nbs to H2-HRP was much stronger than that to fipronil. Since the Nb-magnetosomes from CFFF had the highest binding capability to H2-HRP, they were used for the following studies on the improvement of binding ability to free fipronil.

### Treatment of Nb-magnetosomes with oxidants

**Fig. 4:**
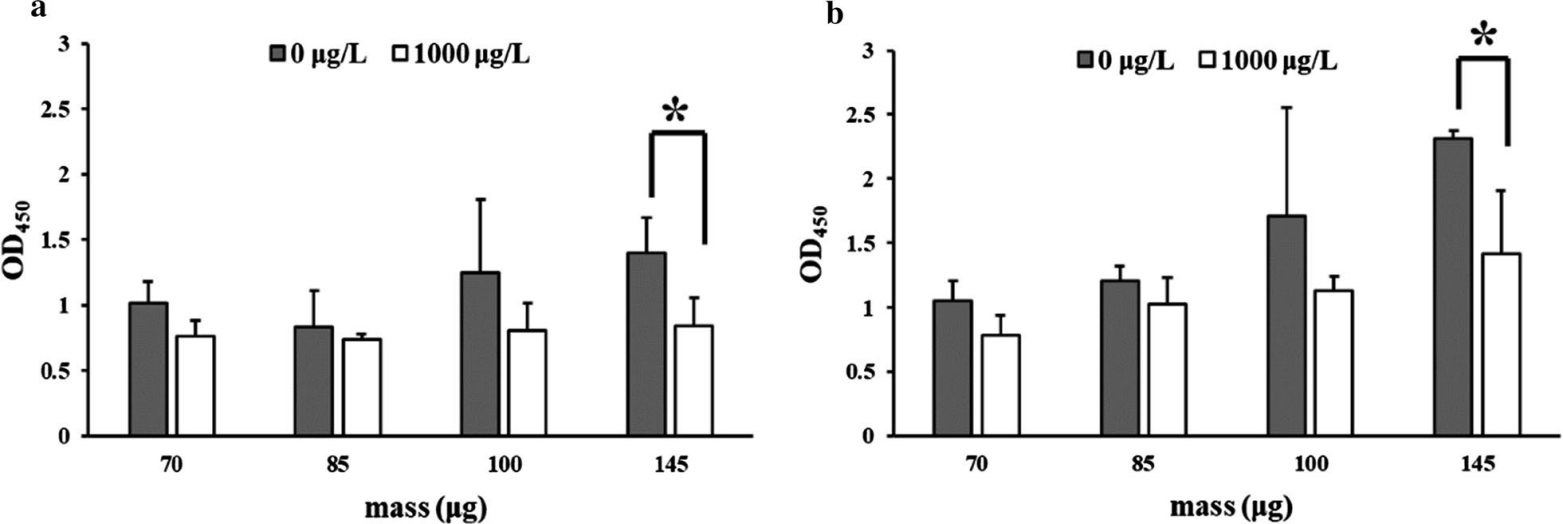
Competitive ELISAs for fipronil using Nb-magnetosomes treated with H_2_O_2_ (**a**) or a mixture of GSH and GSSG (**b**). “*”: significant difference (*p* < 0.05)

Two disulfide bonds might stabilize the three-dimensional structure of the anti-fipronil Nb F1 due to four cysteines existing in the amino acid sequence (Additional file 1: Fig. S3). The formation of disulfide bonds is an oxidative process. Anti-fipronil Nb F1 could, however, be expressed in *E. coli* ER2738 or Top 10F’ with reductive cytoplasms, and they showed high binding capability to fipronil [21]. Nb-magnetosomes were also biomineralized in the reductive cytoplasm media of MSR-1. The failure of Nb-magnetosomes to recognize fipronil may have resulted from the incorrect formation of intramolecular disulfide bonds in a reductive environment in vivo. Oxidation and reduction of disulfide bonds were reported to be an effective means to activate functional proteins in vitro [34, 35]. To improve the binding capability to fipronil, Nb-magnetosomes biomineralized by CFFF were oxidized by H_2_O_2_ (Fig. 4a) or glutathione mixture (GSH and GSSG) in vitro (Fig. 4b). The optimized concentration of H_2_O_2_ was 2% and the optimal ratio of GSH: GSSG in glutathione mixture was 10:1, i.e., the concentrations of GSH and GSSG were 1.0 × 10^–3^ M and 1.0 × 10^–4^ M, respectively. The time treated by H_2_O_2_ and glutathione mixture was 5 and 2 h, respectively.

The binding ability of Nb-magnetosomes to H2-HRP was weakened after oxidation, which the OD_450_ values were lower than those before oxidation. In addition, the OD_450_ was lowered upon H_2_O_2_ treatment (GSH vs GSSG) (Fig. 4). It is noteworthy that the binding capability of all oxidized Nb-magnetosomes to H2-HRP was inhibited in the presence of free fipronil (1000 ng/mL). The maximum inhibition was approximately 40% from the Nb-magnetosomes (145 μg) treated by two oxidants. One of the possible reasons is that after Nb-magnetosomes have been oxidized, the net charge on the nanobodies has changed and disturbed the electrostatic interactions between Nbs and the complex H2-HRP, indirectly improving the binding affinity to fipronil on which the net charge became more suitable for electrostatic interaction. This result is consistent with that of other studies on the modification of protein function via disulfide bond formation in vitro [35, 36].

### Analysis of fipronil in water by ELISA

**Fig. 5:**
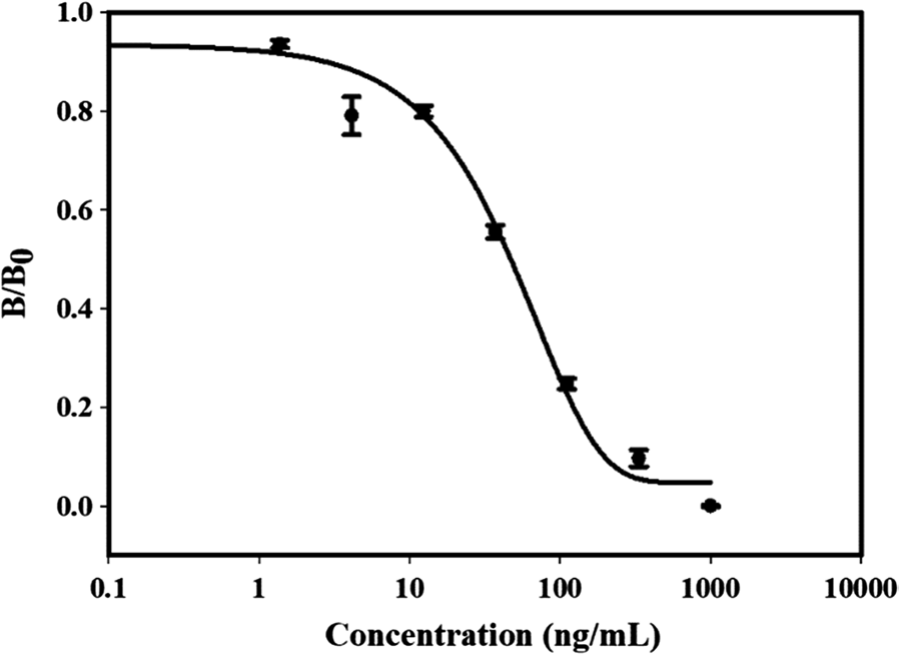
Calibration curve of Nb-magnetosome-based ELISA for fipronil. The data are average of three replicates

The analysis of fipronil by the Nb-magnetosome-based ELISA was optimized and a dose–response curve was generated in 0.01 M PBS (pH 7.4) (Fig. 5), showing a half-maximum signal inhibition concentration (IC_50_) and a limit of detection (LOD, IC_10_) of fipronil at 47 ng/mL and 2.74 ng/mL, respectively. Slight matrix effect of water samples on the assay was observed, but it could be eliminated via sample dilution with PBS at twofold or more than twofold (data not shown). The average recoveries of fipronil from the fortified water samples as determined with ELISAs were in a range of 78–101% (Table 1), illustrating a promise of the oxidized Nb-magnetosomes in immunoassay development for the detection of fipronil in real samples.

**Table 1:** Average recoveries of fipronil from water samples as determined with Nb-magnetosome-based ELISAs

Spiked level (ng/mL)	Detected level (ng/mL) (mean ± SD, n = 3)	Average recovery (CV), %
0	< LOD	
100	100.9 ± 0.01	101 (1.6)
150	116.6 ± 0.03	78 (8.1)
200	192.9 ± 0.01	96 (5.4)

## Discussion

In MTB, approximately 30 specific proteins in the membrane of magnetosomes have been identified so far [7, 9]. Among those proteins, the most abundant one is MamC, followed by MamF. Nbs can be anchored on magnetosomes by genetically fusing to one or more specific membrane proteins. An alpaca-derived Nb specific for RFP was fused with MamC to construct a complex of Nb-magnetosomes, which was used for the immunoprecipitation of RFP-tagged proteins from cell extracts [17]. The three-dimensionality and the high surface-area-to-volume ratio of antibody modified MNPs allowed a relatively high probability of their interaction with targets. Hence, we fused anti-fipronil Nbs with MamC and MamF on magnetosomes for their potential application in various immuno-techniques.

CF was initially constructed by inserting Nb F1 gene into the genome of WT MSR-1, but only low amounts of Nbs were expressed. Then, strain CF+ containing multi-copy F1 genes and strain CFFF with two copy F1 genes were constructed in an attempt to enhance Nb abundances on magnetosomes. For the construction of CF+ , a multi-copy broad host range plasmid pBBRCF containing CF gene was transferred into cytoplasm of CF strain. For CFFF, the suicide plasmid pKFF containing uFFd gene was integrated into the genome of CF strain. Theoretically, the Nbs displayed on magnetosomes from CFFF containing two copies of Nb gene in genome and CF+ containing one copy of Nb gene in genome and another in multi-copies of pBBR1MCS-2 were more than those from CF which has only one copy of Nb gene in genome. However, Nb-magnetosomes biomineralized by the recombinant strains showed different binding capability to H2-HRP in a declining order of CFFF, CF, and CF+ (Fig. 3a). The possible reason is that the expression of genes in the plasmid of CF+ was restricted, leading to the least binding affinity of Nb-magnetosomes from CF+ . The restriction of gene expression in plasmids is not rare, e.g., the construction of a complementary strain with the help of pBBR1MCS-2 was not successful [33].

The formation of disulfide bonds is an oxidative process that generates a covalent bond from two cysteine residues, greatly increasing the stability of proteins (Additional file 1: Fig. S3). Abnormal formation of intramolecular disulfide bonds in MSR-1 may lead to the failure to recognize free fipronil by Nb-magnetosomes. Thereafter, Nb-magnetosomes biosynthesized by CFFF strain were chemically oxidized in vitro. The oxidization by H_2_O_2_ to form disulfide bond has been used in vitro and in vivo [36, 37]. It was reported that two reductive pathways (thioredoxin and glutaredoxin/glutathione) in the cytoplasm would be necessary for the engineering of *E. coli* to produce large quantity of cytoplasmic protein with disulfide bonds [38–42]. By using the thiotransferase glutaredoxin as a repair enzyme and glutathione as cofactor, the reducing potential of NADPH was employed to reduce glutathione via the glutathione oxidoreductase.

In the present study, the binding capability of Nb-magnetosomes to free fipronil was increased after the oxidization by either H_2_O_2_ or the glutathione mixture consisting of GSH and GSSG. Although the sensitivity of the Nb-magnetosome-based ELISA was lower than that of the traditional Nb-based ELISA available in our previous study [21], the former showed some advantages in the cost and assay time (~ 1 h). The oxidized Nb-magnetosomes could be used for versatile applications such as in immunoassays, immunosensors, immunoprecipitation and immunoaffinity chromatography if reductive pathways in cytoplasm of MTB are engineered. It is likely that multiple technological improvements in magnetosome and nanobody engineering will improve incrementally with time making this technology more routine and widely applicable in immunochemical applications.

## Conclusions

In this study, three mutant strains CF, CF+ , and CFFF derived from MSR-1 were constructed by fusion PCR, producing novel functional MNPs consisting of anti-fipronil Nbs and magnetosomes. The Nb-magnetosomes biosynthesized by three mutant strains all recognized H2-HRP but not free fipronil. The Nb-magnetosomes from CFFF oxidized with H_2_O_2_ or GSH/GSSG in vitro have reasonable bind capacity to free fipronil, showing a promise to develop a MNP-based ELISA for the detection of fipronil in water. Furthermore, it was suggested that MTB could be used as a factory to cost-effectively produce eco-friendly Nb-functionalized MNPs that have great prospect in the field of immunochemical science.

## Supplementary Information

**Additional file 1: Table S1.** Bacteria strains and plasmids used in this study. **Table S2.** Primers used in this study. **Fig. S1.** The extraction and purification of Nb-magnetosomes. **Fig. S2.** The structures of fipronil and its hapten (H2). **Fig. S3.** The amino acid sequence of Nb F1.

## References

[1] Chou Chau Y-F, Wang C-K, Shen L, Lim CM, Chiang H-P, Chou Chao C-T, Huang HJ, Lin C-T, Kumara NTRN, Voo NY (2017). Simultaneous realization of high sensing sensitivity and tunability in plasmonic nanostructures arrays. Sci Rep.

[2] Ranmadugala D, Ebrahiminezhad A, Manley-Harris M, Ghasemi Y, Berenjian A (2018). Magnetic immobilization of bacteria using iron oxide nanoparticles. Biotechnol Lett.

[3] Leon-Janampa N, Zimic M, Shinkaruk S, Quispe-Marcatoma J, Gutarra A, Le Bourdon G, Gayot M, Changanaqui K, Gilman RH, Fouquet E, Sheen P, Szlosek M (2020). Synthesis, characterization and bio-functionalization of magnetic nanoparticles to improve the diagnosis of tuberculosis. Nanotechnology.

[4] Gopal J, Abdelhamid HN, Hua P-Y, Wu H-F (2013). Chitosan nanomagnets for effective extraction and sensitive mass spectrometric detection of pathogenic bacterial endotoxin from human urine. J Mater Chem B.

[5] Abdelhamid HN, Wu H-F (2013). Multifunctional graphene magnetic nanosheet decorated with chitosan for highly sensitive detection of pathogenic bacteria. J Mater Chem B.

[6] Lefevre CT, Bennet M, Landau L, Vach P, Pignol D, Bazylinski DA, Frankel RB, Klumpp S, Faivre D (2014). Diversity of magneto-aerotactic behaviors and oxygen sensing mechanisms in cultured magnetotactic bacteria. Biophys J.

[7] Uebe R, Schüler D (2016). Magnetosome biogenesis in magnetotactic bacteria. Nat Rev Microbiol.

[8] Ren E, Lei Z, Wang J, Zhang Y, Liu G (2018). Magnetosome modification: from bio-nano engineering toward nanomedicine. Advanced Therapeutics.

[9] Barber-Zucker S, Zarivach R (2017). A Look into the biochemistry of magnetosome biosynthesis in magnetotactic bacteria. ACS Chem Biol.

[10] Mickoleit F, Lanzloth C, Schüler D (2020). A versatile toolkit for controllable and highly selective multifunctionalization of bacterial magnetic nanoparticles. Small.

[11] Scheffel A, Gardes A, Grunberg K, Wanner G, Schüler D (2008). The major magnetosome proteins MamGFDC are not essential for magnetite biomineralization in *Magnetospirillum gryphiswaldense* but regulate the size of magnetosome crystals. J Bacteriol.

[12] Yoshino T, Matsunaga T (2006). Efficient and stable display of functional proteins on bacterial magnetic particles using Mms13 as a novel anchor molecule. Appl Environ Microbiol.

[13] Kanetsuki Y, Tanaka M, Tanaka T, Matsunaga T, Yoshino T (2012). Effective expression of human proteins on bacterial magnetic particles in an anchor gene deletion mutant of *Magnetospirillum magneticum* AMB-1. Biochem Biophys Res Commun.

[14] Xu J, Liu L, He J, Ma S, Li S, Wang Z, Xu T, Jiang W, Wen Y, Li Y, Tian J, Li F (2019). Engineered magnetosomes fused to functional molecule (protein A) provide a highly effective alternative to commercial immunomagnetic beads. J Nanobiotechnol.

[15] Goldman ER, Liu JL, Zabetakis D, Anderson GP (2017). Enhancing stability of camelid and shark single domain antibodies: an overview. Front Immunol.

[16] Schumacher D, Helma J, Schneider AFL, Leonhardt H, Hackenberger CPR (2018). Nanobodies: chemical functionalization strategies and intracellular applications. Angew Chem Int Ed Engl.

[17] Pollithy A, Romer T, Lang C, Muller FD, Helma J, Leonhardt H, Rothbauer U, Schüler D (2011). Magnetosome expression of functional camelid antibody fragments (nanobodies) in *Magnetospirillum gryphiswaldense*. Appl Environ Microbiol.

[18] Bever CS, Dong JX, Vasylieva N, Barnych B, Cui Y, Xu ZL, Hammock BD, Gee SJ (2016). VHH antibodies: emerging reagents for the analysis of environmental chemicals. Anal Bioanal Chem.

[19] He J, Tian J, Xu J, Wang K, Li J, Gee SJ, Hammock BD, Li QX, Xu T (2018). Strong and oriented conjugation of nanobodies onto magnetosomes for the development of a rapid immunomagnetic assay for the environmental detection of tetrabromobisphenol-A. Anal Bioanal Chem.

[20] He J, Ma S, Wu S, Xu J, Tian J, Li J, Gee SJ, Hammock BD, Li QX (2020). XuT: Construction of immunomagnetic particles with high stability in stringent conditions by site-directed immobilization of multivalent nanobodies onto bacterial magnetic particles for the environmental detection of tetrabromobisphenol-A. Anal Chem.

[21] Wang K, Vasylieva N, Wan D, Eads DA, Yang J, Tretten T, Barnych B, Li J, Li QX, Gee SJ, Hammock BD, Xu T (2019). Quantitative detection of fipronil and fipronil-sulfone in sera of black-tailed prairie dogs and rats after oral exposure to fipronil by camel single-domain antibody-based immunoassays. Anal Chem.

[22] El Hassani AK, Dacher M, Gauthier M, Armengaud C (2005). Effects of sublethal doses of fipronil on the behavior of the honeybee (Apis mellifera). Pharmacol Biochem Behav.

[23] Wright I (2013). Fipronil: a microcosm of flea control?. Companion Anim.

[24] Shi L, Chen L, Wan Y, Zeng H, Xia W (2020). Spatial variation of fipronil and its derivatives in tap water and ground water from China and the fate of them during drinking water treatment in Wuhan, central China. Chemosphere.

[25] Ali I, Peng C, Khan ZM, Naz I (2017). Yield cultivation of magnetotactic bacteria and magnetosomes: a review. J Basic Microbiol.

[26] Rong C, Huang Y, Zhang W, Jiang W, Li Y, Li J (2008). Ferrous iron transport protein B gene (*feoB1*) plays an accessory role in magnetosome formation in *Magnetospirillum gryphiswaldense* strain MSR-1. Res Microbiol.

[27] Zhang Y, Zhang XJ, Jiang W, Li Y, Li J (2011). Semicontinuous culture of *Magnetospirillum gryphiswaldense* MSR-1 cells in an autofermentor by nutrient-balanced and isosmotic feeding strategies. Appl Environ Microbiol.

[28] Wang Q, Liu JX, Zhang WJ, Zhang TW, Yang J, Li Y (2013). Expression patterns of key iron and oxygen metabolism genes during magnetosome formation in *Magnetospirillum gryphiswaldense* MSR-1. FEMS Microbiol Lett.

[29] Schneider P, Hammock BD (2002). Influence of the ELISA format and the hapten-enzyme conjugate on the sensitivity of an immunoassay for S-triazine herbicides using monoclonal antibodies. J Agric Food Chem.

[30] Grant CR, Rahn-Lee L, LeGault KN, Komeili A (2018). Genome editing method for the anaerobic magnetotactic bacterium *Desulfovibrio magneticus* RS-1. Appl Environ Microbiol.

[31] Wang X, Wang Q, Zhang W, Wang Y, Li L, Wen T, Zhang T, Zhang Y, Xu J, Hu J, Li S, Liu L, Liu J, Jiang W, Tian J, Li Y, Schüler D, Wang L, Li J (2014). Complete genome sequence of *Magnetospirillum gryphiswaldense* MSR-1. Genome Announc.

[32] Scheffel A, Gruska M, Faivre D, Linaroudis A, Plitzko JM, Schüler D (2006). An acidic protein aligns magnetosomes along a filamentous structure in magnetotactic bacteria. Nature.

[33] Wang X, Zheng H, Wang Q, Jiang W, Wen Y, Tian J, Sun J, Li Y, Li J (2019). Novel protein Mg2046 regulates magnetosome synthesis in *Magnetospirillum gryphiswaldense* MSR-1 by modulating a proper redox status. Front Microbiol.

[34] Eggenreich B, Willim M, Wurm DJ, Herwig C, Spadiut O (2016). Production strategies for active heme-containing peroxidases from *E. coli* inclusion bodies - a review. Biotechnol Rep (Amst).

[35] Carroll L, Jiang S, Irnstorfer J, Beneyto S, Ignasiak MT, Rasmussen LM, Rogowska-Wrzesinska A, Davies MJ (2020). Oxidant-induced glutathionylation at protein disulfide bonds. Free Radic Biol Med.

[36] Karala AR, Lappi AK, Saaranen MJ, Ruddock LW (2009). Efficient peroxide-mediated oxidative refolding of a protein at physiological pH and implications for oxidative folding in the endoplasmic reticulum. Antioxid Redox Signal.

[37] Margittai E, Low P, Stiller I, Greco A, Garcia-Manteiga JM, Pengo N, Benedetti A, Sitia R, Banhegyi G (2012). Production of H_2_O_2_ in the endoplasmic reticulum promotes in vivo disulfide bond formation. Antioxid Redox Signal.

[38] Lobstein J, Emrich CA, Jeans C, Faulkner M, Riggs P, Berkmen M (2012). SHuffle, a novel* Escherichia col*i protein expression strain capable of correctly folding disulfide bonded proteins in its cytoplasm. Microb Cell Fact.

[39] Derman AI, Prinz WA, Belin D, Beckwith J (1993). Mutations that allow disulfide bond formation in the cytoplasm of *Escherichia coli*. Science.

[40] Stewart EJ, Aslund F, Beckwith J (1998). Disulfide bond formation in the *Escherichia coli* cytoplasm: an in vivo role reversal for the thioredoxins. EMBO J.

[41] Ritz D, Lim J, Reynolds CM, Poole LB, Beckwith J (2001). Conversion of a peroxiredoxin into a disulfide reductase by a triplet repeat expansion. Science.

[42] Bessette PH, Aslund F, Beckwith J, Georgiou G (1999). Efficient folding of proteins with multiple disulfide bonds in the *Escherichia coli* cytoplasm. Proc Natl Acad Sci U S A.

