# Supplementary material for: Fusion expression of nanobodies specific for the insecticide fipronil on magnetosomes in Magnetospirillum gryphiswaldense MSR-1
Source: J Nanobiotechnology. 2021 Jan 19;19:27. doi: 10.1186/s12951-021-00773-z (PMC7816308; doi:10.1186/s12951-021-00773-z)
Supplement: Supplementary file 1 — Additional file 1: Table S1. Bacteria strains and plasmids used in this study. Table S2. Primers used in this study. Fig. S1. The extraction and purification of Nb-magnetosomes. Fig. S2. The structures of fipronil and its hapten (H2). Fig. S3. The amino acid sequence of Nb F1. [file 12951_2021_773_MOESM1_ESM.docx]

**Fusion expression of nanobodies specific for the insecticide fipronil on magnetosomes in *Magnetospirillum gryphiswaldense* MSR-1**

Sha Wu^1, 2^, Fengfei Ma^1, 2^, Jinxin He^1, 2^, Qing X. Li^3^, Bruce D. Hammock^4^, Jiesheng Tian^5^*, Ting Xu^1, 2^*

^1^ Beijing Key Laboratory of Biodiversity and Organic Farming, College of Resources and Environmental Sciences, China Agricultural University, Beijing 100193, China

^2^ Suzhou Vicheck Biotechnology Co. Ltd., Suzhou 215128, China

^3^ Department of Molecular Biosciences and Bioengineering, University of Hawaii at Manoa, 1955 East-West Road, Honolulu, Hawaii 96822, USA

^4^ Department of Entomology and Nematology and UCD Comprehensive Cancer Center, University of California, Davis, California 95616, United States

^5^ Department of Microbiology, College of Biological Sciences, China Agricultural University, Beijing 100193, China

*Corresponding author:

E-mail: tianhome@cau.edu.cn. Tel: +86-10-62733751

E-mail: [xuting@cau.edu.cn](mailto:xuting@cau.edu.cn). Tel: +86-10-62733482, Fax: +86-10-62732498

Table S1. Bacteria strains and plasmids used in this study

| Name | | Genotype or relevant characteristic | source |
| --- | --- | --- | --- |
| *E.coli* | DH5α | *endA1 hsdR17*[r-m+] *supE44 thi-1 recA1 gyrA*[NalR] *relA relA1* ∆[*lacZYA-argF*] *U169 deoR* [Ø80∆(*LacZ*) M15] | Novagen |
|  | S17-1 | *Thi endA recA hsdR* with RP4-2Tc::Mu-Km::Tn7 integrated in chromosome; Sm^r^, Tra^+^ | Novagen |
| *M. gryphiswaldense* | MSR-1 WT | Wild type *Magnetospirillum gryphiswaldense*：Nx^r^ | DSM6361 |
|  | CF | Substitute CF for *mamC* in MSR-1 WT genome: Nx^r^ | This study |
|  | CFFF | inserting FF into mutant strain CF genome: Nx^r^, Kan^r^ | This study |
|  | CF+ | CF mutant strain harboring pBBRCF: Nx^r^, Kan^r^ | This study |
| Plasmids | pMD^TM^ 19（simple） | Cloning vector: Amp^r^ | Takara |
|  | pK18mobSacB | Suicide vector for MSR-1: Kan^r^, sucrose^r^ | This study |
|  | pKCF | pK18mobSacB containing fusing gene consisting with fipronil VHH, *mamC*, and *mamC* flanking sequences: Kan^r^, sucrose^r^ | This study |
|  | pKFF | pK18mobSacB containing fusing gene consisting with fipronil VHH, *mamF*, and *mamF* flanking sequences: Kan^r^, sucrose^r^ | This study |
|  | pBBR1MCS-2 | Multi-copy broad host range vector：Kan^r^ | This study |
|  | pBBRCF | pBBR1MCS-2 containing fusing gene consisting of fipronil VHH and *mamC*: Kan^r^ | This study |

Table S2. Primers used in this study

| Name | Sequence (5’→3’) | Description |
| --- | --- | --- |
| MamC-F (*EcoR* I) | **CCG*GAATTC*CGG**ATTAAGGACAACAGCGAT | CF or FF gene |
| MamC-R | GGAACCGCCGCCACCAGAGCCACCACCGGCGGAGGCCAATTCTTCCCTCAG |  |
| Fip-F | GGCTCTGGTGGCGGCGGTTCCGGTGGCGGTGGCAAAAAGACAGCTATCGCG |  |
| Fip-R (*Xba* I) | **GC*TCTAGA*GC**TCACTTGCATACTTCATT |  |
| MamF-F | ATGGCCGAGACTATTTTG |  |
| MamF-R | GGAACCGCCGCCACCAGAGCCACCACCGGCGGAGATCAGGGCGACTACATG |  |
| mamC-F | ATTAAGGACAACAGCGAT | CF containing *mamC*’s flanking sequences |
| Fip-R | TCACTTGCATACTTCAT |  |
| Cu-F(*EcoR* I) | **CCG*GAATTC***GGGTAAGAGCTTCATCGT |  |
| Cu-R | CTCATCGCTGTTGTCCTT |  |
| Cd-F | AATGAAGTATGCAAGTGAAATATTGGGCTGGTTCAC |  |
| Cd-R(*Xba* I) | **GC*TCTAGA***CCACAAAGTA CCGAGACC |  |
| Fu-F(*EcoR* I) | **CCG*GAATTC***CCTTAACGCCAATGACCACCAC | *mamF*’s flanking sequences |
| Fu-R | CGATCAAAATAGTCTCGGCCAT |  |
| Fd-F | AATGAAGTATGCAAGTGACGGCGAGCGATCTAACGGAC |  |
| Fd-R(*Xba* I) | **GC*TCTAGA***AGGTATAAAGCGCGACAACAC |  |
| P1 | AACTGGATTGCCTTGACC |  |
| P2 | CCGCTCTAATCTGCTATC |  |
| P3 | CAGCTAGCACCGTACACA |  |
| P4 | TGTGTACGGTGCTAGCTG |  |
| pBBR-F | GCGTAATACGACTCACTAT |  |
| pBBR-R | TTAACCCTCACTAAAGGGA |  |

Note: italic, bold and underline font: restriction enzyme sequence; bold and underline font: protection bases of restriction enzyme; box: PCR fusing region sequence; “F”: forward; “R”: reverse; all primers were synthesized by ThermoFisher.


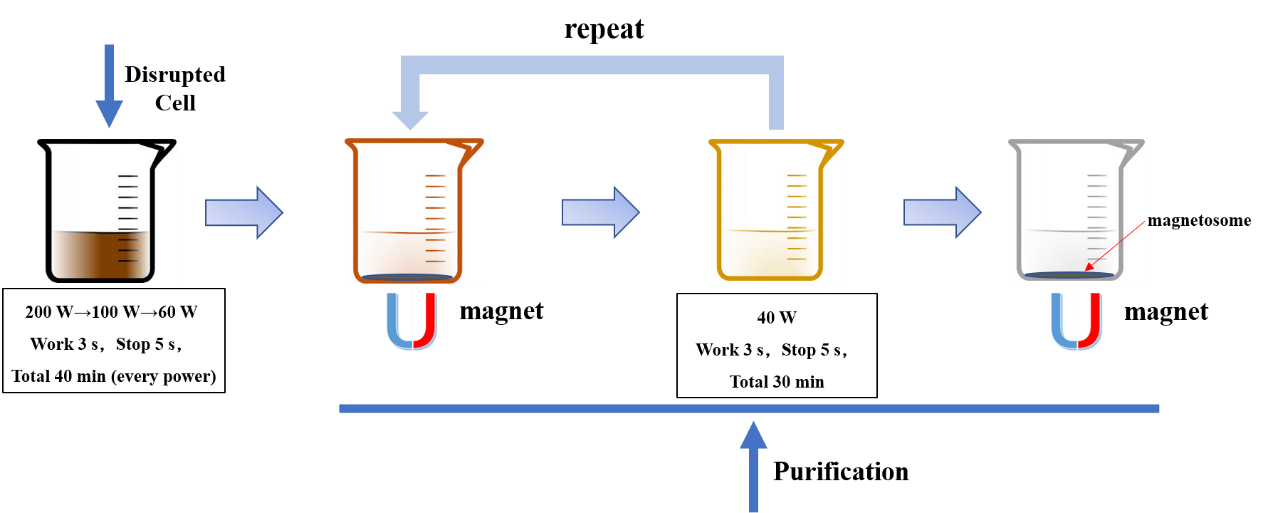


Fig. S1. The extraction and purification of Nb-magnetosomes


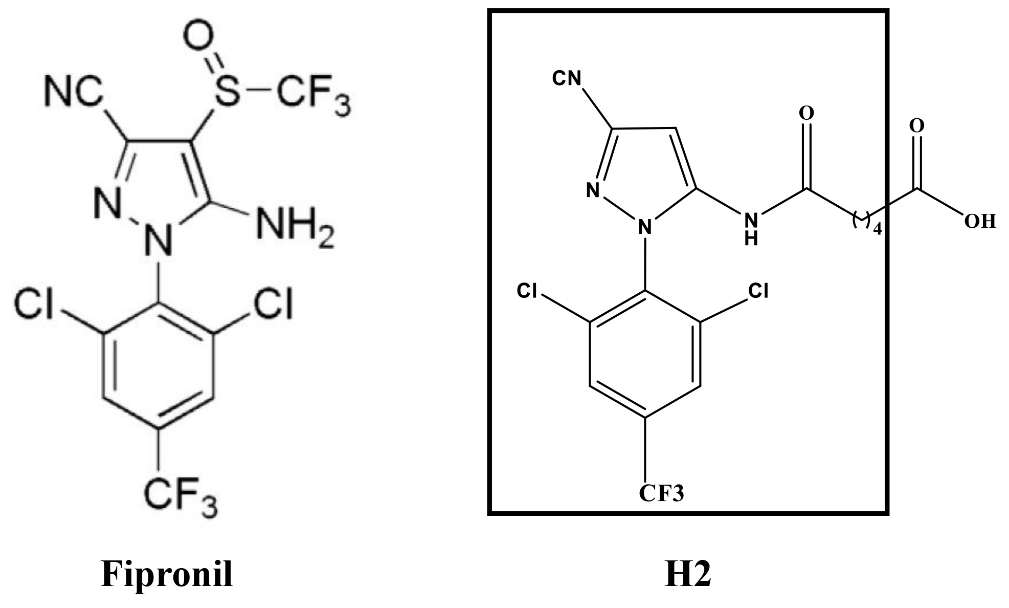


Fig. S2. The structures of fipronil and its hapten (H2)

Fig. S3. The amino acid sequence of Nb F1.
